# Size matters: predation of fish eggs and larvae by native and invasive amphipods

**DOI:** 10.1007/s10530-016-1265-4

**Published:** 2016-09-08

**Authors:** N. G. Taylor, A. M. Dunn

**Affiliations:** School of Biology and water@leeds, University of Leeds, Leeds, LS2 9JT UK

**Keywords:** *Dikerogammarus*, Predatory functional response, Invasive species, Impact, Body size

## Abstract

Invasive predators can have dramatic impacts on invaded communities. Extreme declines in macroinvertebrate populations often follow killer shrimp (*Dikerogammarus villosus*) invasions. There are concerns over similar impacts on fish through predation of eggs and larvae, but these remain poorly quantified. We compare the predatory impact of invasive and native amphipods (*D. villosus* and *Gammarus pulex*) on fish eggs and larvae (ghost carp *Cyprinus carpio* and brown trout *Salmo trutta*) in the laboratory. We use size-matched amphipods, as well as larger *D. villosus* reflecting natural sizes. We quantify functional responses, and electivity amongst eggs or larvae and alternative food items (invertebrate, plant and decaying leaf). *D. villosus*, especially large individuals, were more likely than *G. pulex* to kill trout larvae. However, the magnitude of predation was low (seldom more than one larva killed over 48 h). Trout eggs were very rarely killed. In contrast, carp eggs and larvae were readily killed and consumed by all amphipod groups. Large *D. villosus* had maximum feeding rates 1.6–2.0 times higher than the smaller amphipods, whose functional responses did not differ. In electivity experiments with carp eggs, large *D. villosus* consumed the most eggs and the most food in total. However, in experiments with larvae, consumption did not differ between amphipod groups. Overall, our data suggest *D. villosus* will have a greater predatory impact on fish populations than *G. pulex*, primarily due to its larger size. Higher invader abundance could amplify this difference. The additional predatory pressure could reduce recruitment into fish populations.

**Electronic supplementary material:**

The online version of this article (doi:10.1007/s10530-016-1265-4) contains supplementary material, which is available to authorized users.

## Introduction

Alien invasive species continue to have negative impacts on populations, communities and ecosystems across the globe (Strayer 2010; Simberloff et al. 2013; Gallardo et al. 2016). One important mechanism behind these impacts is predation (Ross 1991; Mack et al. 2000; Davis 2003; Sax and Gaines 2008; Kumschick et al. 2012; Blackburn et al. 2014). Predation is a fundamental ecological interaction with the capacity to shape and structure natural communities (Thorp 1986; Case and Bolger 1991; Wellborn et al. 1996; Jackson et al. 2001). Owing to factors such naivety in prey populations (Case and Bolger 1991; Cox and Lima 2006), release from natural enemies (Roy et al. 2011) or intrinsic behavioural characteristics (Weis 2010), invasive predators frequently consume prey more rapidly than analogous native species and thus have stronger effects on resident prey populations (Dick et al. 2014).

Invasive species are one of the primary threats to freshwater biodiversity, reflecting the globally extensive but locally intensive use of fresh waters by humans (Richter et al. 1997; Sala et al. 2000; Millennium Ecosystem Assessment 2005; Dextrase and Mandrak 2006; Light and Marchetti 2007). Moreover, introduced predators in freshwaters have particularly severe impacts relative to those in terrestrial or marine systems (Sala et al. 2000; Cox and Lima 2006). For example, fish populations—many of great commercial or biological importance—frequently decline following invasion as a result of predation. All life stages are vulnerable, from adults (e.g. Lawrie 1970; Ogutu-Ohwayo 1990; Ruzycki et al. 2003) to young fish (e.g. Garman and Nielsen 1982; Lemly 1985) to eggs and larvae (e.g. Meffe 1985; Ruzycki et al. 2003).

Predation is probably the biggest single cause of fish egg and larval mortality (Bailey and Houde 1989; Houde 2002). Consequently, it can have particularly strong effects on populations, greatly influencing recruitment of even the most fecund fish (Köster and Möllmann 2000; Bajer et al. 2012). For example, in experimental ponds, egg predation by *Orconectes virilis* decreased or completely prevented recruitment of pumpkinseed (*Lepomis gibbosus*) and bluegill (*L. macrochirus*) sunfish respectively (Dorn and Mittelbach 2004). Meanwhile, in the Upper Mississippi River Basin, egg predation by *L. macrochirus* drastically reduces carp recruitment, providing local biotic resistance to invasion by carp where the predator is present (Bajer et al. 2012). Vulnerability to predation is conferred by the aggregated distribution and limited mobility of fish eggs and larvae (Hassell 1978; McGurk 1986). Moreover, their small size makes them accessible to a wide range of predators, including macroinvertebrates such as Trichoptera, Plecoptera and Crustacea (Zuromska 1966; Fox 1978; Mills 1981; Brown and Diamond 1984).

The amphipod crustacean *Dikerogammarus villosus* (Sowinsky 1894) is a potentially devastating invasive predator of fish eggs and larvae. *D. villosus* is native to the Ponto-Caspian region, but is spreading north-west through the river and canal network of Europe (Bij de Vaate et al. 2002; Gallardo et al. 2012; Rewicz et al. 2014) and threatens to invade elsewhere (e.g. the American Great Lakes; Pagnucco et al. 2014). Evidence implicates *D. villosus* as a voracious predator, earning it the ‘killer shrimp’ title, special attention as an ‘alert’ species in Great Britain, and a listing as one of the 100 worst invaders in Europe (Delivering Alien Invasive Species in Europe project www.europe-aliens.org).

Invasion by *D. villosus* frequently coincides with the decline or extinction of resident benthic macroinvertebrates such as isopods, tubificids and amphipods (Dick and Platvoet 2000; Dick et al. 2002; Kley and Maier 2003; Josens et al. 2005; Boets et al. 2010; MacNeil et al. 2013; Dodd et al. 2014; Gergs and Rothhaupt 2015). Thus, once established *D. villosus* typically dominates the macroinvertebrate community in both number and biomass (Josens et al. 2005; van Riel et al. 2006). Trophic links and ecosystem functions can also be transformed by the invader (Dick et al. 2002; Piscart et al. 2011; MacNeil et al. 2011; Boeker and Geist 2015). Predation by *D. villosus* may be an important mechanism behind these changes. In the laboratory, *D. villosus* will consume a wide range of animal prey, including aquatic bugs, leeches, isopods, juvenile crayfish, chironomid larvae, odonate larvae, ephemeropteran larvae and even other amphipods (Dick and Platvoet 2000; Platvoet et al. 2009; Boets et al. 2010; MacNeil et al. 2013). Stable isotope and fatty acid analyses suggest predatory tendencies tend to be retained in the field (van Riel et al. 2006; Maazouzi et al. 2007; but see Hellmann et al. 2015).

*D. villosus* will also prey upon fish eggs and larvae, raising concerns about its potential to cause analogous declines in fish populations. *D. villosus* will kill and eat *Cottus perifretum* eggs and larvae in the laboratory and have been found with damaged *C. perifretum* eggs in the field (Platvoet et al. 2009). Further, Casellato et al. (2007) showed that *D. villosus* will consume *Coregonum lavaretus* eggs preferentially over other animal prey. However, these experiments produce few quantitative data for few species of fish, and do not compare impacts with native species. Comprehensive and objective data on invader impacts, ideally relative to native species, are vital to understand how invaders might change ecosystems and as a basis for management decisions (Byers et al. 2002; NRC 2002; Kumschick et al. 2012; Dick et al. 2013, 2014).

Using laboratory experiments, we compare predatory impacts of invasive *D. villosus* and an analogue native to Great Britain, *Gammarus pulex* (L. 1758), on the early life stages of salmonid and coarse (i.e. non-salmonid) fish. We use size-matched amphipods to examine intrinsic differences between species as well as large *D. villosus* to reflect natural differences in amphipod size: both species identity and body size can be critical aspects of predator–prey interactions (Bailey and Houde 1989; Luecke et al. 1990; Miller et al. 1992; Woodward et al. 2005; Rall et al. 2012; Rudolf et al. 2014; Anderson et al. 2016). We quantify amphipod predation on fish eggs and larvae (a) as functional responses (FRs), a fundamental measure of resource use with the potential to predict impacts in the field (Dick et al. 2013, 2014) and (b) in the presence of alternative foods to examine differences in electivity, which can also influence predator impacts (Grosholz 2005; Dodd et al. 2014). Finally, we discuss the results of these experiments in the context of potential impacts on fish populations.

Since damaging invasive species tend to consume resources at faster rates than native analogues (Dick et al. 2014), we predict that *D. villosus* will have a higher FR and consume more food in electivity experiments than size-matched *G. pulex*. We also predict larger *D. villosus* will consume more food than the smaller amphipods in both FR and electivity experiments (Woodward et al. 2005; Maier et al. 2011; Rall et al. 2012). In electivity experiments, we predict that *D. villosus* will show a stronger tendency than *G. pulex* to consume fish eggs and larvae given the known predatory tendencies of the invader (e.g. van Riel et al. 2006).

## Methods

### Experimental organisms

#### Fish eggs and larvae

Fish were a representative salmonid (native brown trout *S. trutta* L. 1758) and coarse fish (non-native ghost carp *Cyprinus carpio* L. 1758). These were chosen to represent two contrasting sizes of freshwater fish propagule (Table 1; Teletchea and Fontaine 2010), the two main types of freshwater fishery in the UK (Mawle and Peirson 2009) and the most speciose European fish families (Freyhof and Brooks 2011).

**Table 1:** Length and mass of fish eggs and larvae used in experiments. *n* = 24, except for trout eggs *n* = 10

Fish	Stage	Length (mm)	SE	Mass (mg)	SE
Carp	Egg	1.92	0.01	3.81	0.07
	Larva	5.69	0.07	1.32	0.06
Trout	Egg	5.04	0.05	70.60	1.51
	Larva	15.37	0.24	65.60	1.46

Carp larvae were measured after killing in 70 % ethanol

Live trout eggs were sourced from a commercial hatchery in Grassington, UK in January and kept in aerated, aged and circulating tap water in incubators at 7.0 ± 0.2 °C (range) and under a 9:15 h light:dark cycle. Live carp eggs were sourced from a commercial hatchery in Nottingham, UK in early May and kept in aerated, aged and circulating tap water in a controlled-temperature (CT) room at a temperature of 13.9 ± 0.1 °C (range) and under a 12:12 h light:dark cycle. Temperatures and light regimes were chosen to match typical development conditions for each fish (Alabaster and Lloyd 1982). Tap water was aged (at the same temperature as the eggs) through continual aeration in plastic jerry cans for 24 h. Egg and larval stock tanks were cleaned daily, with conditions adequate to yield high survival and hatch rates. Larvae were only kept and used when recently-hatched and relying on yolk sacs for nutrition (Teletchea and Fontaine 2010), thus falling outside the remit of the UK Animals (Scientific Procedures) Act (1986). Mean sizes of eggs and larvae (Table 1) were typical for salmonids and coarse fish (Teletchea and Fontaine 2010).

#### Amphipods

*G. pulex* were kick-sampled from a stream in Golden Acre Park, Leeds (lat 53°52′N, long 1°36′W) and *D. villosus* sampled from artificial substrates in Grafham Water, Cambridgeshire (lat 52°17′N, long 0°19′W). Each species was transported to Leeds in insulated boxes and maintained in the laboratory on a diet of stream-conditioned *Acer pseudoplatanus* L. leaves (which were readily consumed). Amphipods were kept in aerated, aged tap water under the same light and temperature regime as fish eggs and larvae for at least 1 week before use in experiments, and in single-sex tanks for at least 72 h before use.

Only male amphipods were used in experiments to avoid potential variation in predatory impact with breeding status in females, and control for the fact that male *D. villosus* may be more predatory than females (Dick and Platvoet 2000; Kinzler and Maier 2003). Males were identified by precopulatory pairing (*G. pulex*) or presence of genital papillae and absence of oostegites (*D. villosus*). All amphipods were free of obvious visual parasites that may affect behaviour (Dick et al. 2010; Bacela-Spychalska et al. 2013). Amphipods were only used once in each experiment (i.e. combination of fish species, developmental stage and experimental design) but were re-used between experiments within fish species. Re-used amphipods always had at least 24 h to recover in communal tanks, and all amphipods had the same level of experience with prey at the start of each experiment.

Following Dodd et al. (2014) and in recognition of the larger size of *D. villosus* (pers. obs.; Pinkster 1970; Nesemann et al. 1995; Kinzler et al. 2009) amphipods were divided into three size groups: large *G. pulex,* intermediate *D. villosus* and large *D. villosus*. Amphipods were size-matched by eye prior to experiments, keeping handling and stress to a minimum. On termination of experiments, amphipods were weighed (live, blotted dry) and photographed (in curved natural resting state), with length subsequently measured as a curved line from rostrum tip to telson tip in ImageJ (Rasband 1997–2016). Datasets for all experiments were rarefied using post-experiment body size parameters to ensure size-matching between large *G. pulex* and intermediate *D. villosus*, thus allowing comparison of intrinsic differences in the species’ predatory impact. Meanwhile, large *D. villosus* were significantly longer and heavier than intermediate *D. villosus* and large *G. pulex* in all experiments, enabling quantification of differences in predation rate associated with the larger size of the invader. Mean lengths and masses of amphipod groups used in each experiment, and statistical comparisons, are given in Section S1 (Supplementary Information). Mean sizes (±SE) across all experiments were: large *G. pulex* length 16.54 ± 0.08 mm, mass 46.95 ± 0.57 mg; intermediate *D. villosus* 16.79 ± 0.11 mm, 48.81 ± 0.70 mg; and large *D. villosus* 22.12 ± 0.09 mm, 106.72 ± 1.12 mg.

### Functional response (FR) experiments

#### Experimental design

Four separate experiments were run in which amphipods were presented with a single prey type (carp eggs or larvae, or trout eggs or larvae) in varying densities—one experiment for each prey type. The aim of these experiments was to quantify predator FRs, modelling the relationship between resource use and availability (Holling 1959; Dick et al. 2013). This methodology for comparing invasive and native species’ impacts is becoming widely adopted and is accumulating supporting evidence (Haddaway et al. 2012; Dick et al. 2013; Alexander et al. 2014; Paterson et al. 2014; Dick et al. 2014).

Individual amphipods were starved for 24 h, in clear plastic arenas (87 mm diameter, 50 mm depth) with approximately 200 ml of aged tap water and a single glass bead (20 mm diameter, 9 mm height) as substrate to prevent perpetual swimming. Starved amphipods were then transferred to experimental arenas, identical to starvation conditions but containing a known number of prey items (1, 2, 3, 5, 8, 10, 15, 25, 35, 50 or 80 carp eggs; 1, 2, 3, 5, 8, 12, 25 or 50 carp larvae; or 1, 3, 5, 8, 12, 16, 25, 35 or 50 trout eggs or larvae). Egg membrane strength (Zotin 1958) and larval swimming ability (Fuiman 2002) change over time, but we only selected eggs that were robust on handling, only used larvae >12 h (carp) or >24 h (trout) old, and observed no obvious changes in larval swimming ability over the time course of the experiments. Furthermore, treatments (amphipod group x density combinations) were blocked by day within each experiment to control for any temporal variation in prey (and predator) condition. Within each block, arenas were randomly arranged in space. Controls (without an amphipod) were run at all prey densities to check prey survival in the absence of predators. Controls were interspersed spatially and temporally with experimental arenas.

Arenas were placed in incubators with temperature and light regimes identical to those used to keep stock eggs and larvae: 13.9 ± 0.1 °C (range) with 12:12 h light:dark cycle for carp, and 7.0 ± 0.2 °C (range) with 9:15 h light:dark cycle for trout. Temperatures were within the range at which both amphipod species will feed (Sutcliffe et al. 1981; van der Velde et al. 2009; Maier et al. 2011). Each amphipod was allowed to feed for a set period: 24 h on carp eggs or larvae, or 48 h on trout because preliminary experiments indicated that predation rates on trout were much lower.

At the end of this experimental period, amphipods were removed and remaining alive, dead and damaged prey (body parts) enumerated. For each damaged prey item, the amount of flesh remaining was estimated by eye, to the nearest 10 %. Consumption was calculated as the number of prey supplied minus all remaining flesh (whole and damaged prey). Deaths due to predation were defined as prey that had been wholly or partially consumed, as opposed to dead but undamaged prey assumed to reflect background mortality (≤3.2 % in all experiments). The number of partially consumed larvae was estimated from remaining body parts, assuming that if two body parts may have originated from a single individual (e.g. a tail and a head) then they did so.

Used amphipods were isolated, fed with conditioned *A. pseudoplatanus* leaves and monitored for 24 h. Any individuals that moulted or died in this period were excluded from our dataset. Following rarefaction to ensure size-matching, data were retained for at least four replicates at all prey densities and at least five replicates (and up to eight) for densities of five or more.

#### Statistical methods

All statistical analyses were carried out in R version 3.2.1 (R Core Team 2015) with α = 0.05.

For the experiments with carp eggs and larvae, predation was sufficient to construct and compare FR curves. Analyses were carried out using number of prey consumed (rounded to the nearest whole prey) or number of prey killed as response variables, but for carp prey we present only the former in the main text (a) to be consistent with analyses of electivity experiments and (b) because partial consumption was rare, so consumption was closely associated with number of prey killed and thus a reasonable basis for predicting population impacts. If frequent, partial consumption could decouple this consumption-impact relationship (Dick et al. 2002).

To determine FR type, the relationship between proportional consumption of prey and prey density was modelled using second order logistic regression with quasibinomial error distributions to account for overdispersion (Crawley 2007). The sign and significance of the coefficients indicate FR type (Trexler et al. 1988; Juliano 2001).

Then, FRs were modelled using Rogers’ random predator equation [Eq. (1), Rogers 1972], appropriate because FRs were Type II and prey were not replaced over the course of the experiments (Juliano 2001).

1 $${N_e} = {N_0}\left( {1 - \exp \left( {a\left( {{N_e}h -\text{T}} \right)} \right)} \right)$$

where *N*_*e*_ is the number of prey eaten, *N*_0_ is the initial density of prey, *a* is the attack coefficient, *h* is the handling time and T is the total time available for predation (days). Modelling was performed in the R package *frair* (Pritchard 2014), which utilises maximum likelihood estimation within the *bbmle* package (Bolker 2014) and a modified version of Eq. (1) with an additional *Lambert W* function to make the equation solvable (Eq. (2)).

2 $${N_e} = {N_0} - \hbox{lambert}W\left( {a\cdot h\cdot{N_0}\cdot\exp \left( { - a\left( {\hbox{T} - {N_0}h} \right)} \right)} \right)/\left( {a\cdot h} \right)$$

Curves were bootstrapped to visualise variability (*n* = 1999), and the parameters *a* and *h* compared between amphipod groups (within each prey type) and prey types (within amphipod groups) using indicator variables (function *frair_compare*; Juliano 2001; Paterson et al. 2014).

Incidence of partial consumption of carp larvae (whether individual amphipods partially consumed any carp larvae) was analysed with respect to prey density and amphipod group using a generalised linear model (GLM) with binomial errors. Then, considering just amphipods that exhibited partial consumption, the number and proportion of partially consumed larvae were analysed with respect to prey density and amphipod group using GLMs, with quasipoisson and quasibinomial errors respectively. To identify significant explanatory variables, GLMs were simplified to minimum adequate models (MAMs) following Crawley (2007), discarding terms whose exclusion from the model did not significantly increase deviance. χ^2^ tests of significance were employed for binomial models, and *F* tests of significance for models involving quasi-likelihood.

In FR experiments with trout eggs, negligible levels of predation precluded statistical analysis. In FR experiments with trout larvae, levels of predation were too low to fit FR curves. Instead, incidence of predation (whether individual amphipods killed any larvae) was analysed with respect to prey density and amphipod group using a GLM with binomial errors, simplified as above (Crawley 2007). Then, amongst the amphipods that killed larvae, the magnitude of predation (number of larvae killed) and incidence of partial consumption were analysed with respect to prey density and amphipod group through simplification of quasipoisson and binomial GLMs respectively. Finally, the amount of flesh consumed by predators was compared between amphipod groups using Kruskal–Wallis tests with post hoc Dunn tests (package *dunn.test*; Dinno 2016) and Holm-Bonferroni adjustment of *p* values (Holm 1979).

### Electivity experiments

#### Experimental design

Predatory impact also depends on electivity: the relative proportions of food types in a consumer’s diet compared with the relative proportions available (Ivlev 1961; Underwood et al. 2004). Electivity is a similar concept to preference, but does not imply behavioural choices by the consumer that were unquantified in this study. Here, we quantified amphipod electivity in two experiments—one involving carp eggs with three alternative food types, and one involving carp larvae with three alternative food types—with particular focus on the tendency of amphipods to consume eggs and larvae in the presence of alternative foods.

Alternative food types were selected based on likely coincidence with carp eggs and larvae, and on prior knowledge of consumption by gammarids (Eichenberger and Weilenmann 1982; MacNeil et al. 1997; Platvoet et al. 2009). Plants were fresh, live *Ranunculus aquatilis* L. (ordered online). Leaves were *A. pseudoplatanus* leaf discs, 1 cm diameter (leaves collected from Woodhouse Ridge, Leeds, lat 53°52′N, long 1°36′W, and conditioned in stream water for three months). Invertebrates were *Asellus aquaticus* (L. 1758) isopods (collected from Woodhouse Ridge, Leeds).

Arenas were set up containing 180 ml of aged tap water, fifteen glass beads (20 mm diameter, 9 mm height) to provide habitat structure, and four food types: 10 carp eggs or larvae, plus 3–5 leaf discs, 1–3 *R. aquatilis* sections and 2–3 live *A. aquaticus*. Most food types were presented in approximately equal masses (range 34–47 mg across all arenas but <10 % variation in mass between food types within each arena). However, because of their small size (Table 1), adding a similar mass of carp larvae would have made them unrealistically abundant. Larvae were also too fragile to weigh prior to experiments. Thus, 10 carp larvae were added to each arena, to match the number of eggs presented in prior experiments with eggs. Food was generally provided in excess (<30 % total mass was consumed and no individual food type completely was consumed, except for larvae in four of twelve arenas containing *G. pulex*).

Individual amphipods (starved for 24 h as for FR experiments) were transferred to experimental arenas and allowed to feed for 24 h. Environmental conditions in incubators were the same as for carp stocks: 13.9 ± 0.1 °C (range) with 12:12 h light:dark cycle. Within each experiment, treatments (amphipod groups) were blocked by day, and within each block arenas were randomly arranged in space. Controls (arenas with four food types but no amphipod, to quantify prey survival and autogenic change in food masses) were interspersed spatially and temporally with experimental arenas.

At the end of the feeding period, amphipods were removed from their arena. Remaining food items were counted and, except for larvae, weighed to the nearest mg. For larvae, approximate initial and final masses were back-calculated from the mean mass of a separate sample of larvae (Table 1). Used amphipods were monitored for 24 h as for FR experiments. Data for amphipods that died or moulted in this period were removed, leaving a final data set with 9–15 replicates for each amphipod group in each experiment.

#### Statistical methods

A small amount of autogenic change was observed in food choice controls (mean ± SE change in mass: carp eggs −0.3 ± 0.4 mg; leaf discs −1.8 ± 0.4 mg; *R. aquatilis* +1.7 ± 0.3 mg; *A. aquaticus* −1.9 ± 0.7 mg; carp larvae not weighed). Thus, true consumption was calculated by adjusting masses consumed in the presence of an amphipod by the change in mass in their absence (Haddaway et al. 2012).

First, the mass of eggs, larvae and all food consumed in each experiment were compared between amphipod groups. Where residuals were normal (after log transformation where necessary), ANOVA and post hoc Tukey HSD tests were used to compare means. Zeros in the *G. pulex* egg consumption data rendered parametric tests unsuitable, so egg consumption was compared using a Kruskal–Wallis test and post hoc Dunn tests (Dinno 2016) with step-down Holm-Bonferroni adjustment of *p* values (Holm 1979).

Second, within each experiment and amphipod group, compositional analysis was used to detect non-random feeding and rank food items by their contribution to amphipod diet. Although originally proposed as a method to compare habitat usage, compositional analysis can equally be applied to diets (Aebischer et al. 1993; Brickle and Harper 1999; Anderson et al. 2000; Strain et al. 2014).

The diet composition of each individual amphipod was summarised as the percentage contribution of each food type (fish, leaf, plant or invertebrate) to total mass consumed. Availability was defined as the percentage mass of each food presented (analyses assuming equal availability in the larvae experiments generated identical rankings; Table S5). These data were analysed the R package *adehabitatHS* (Calenge 2015), which first converts the percentages into log-ratios, making data for each food group linearly independent and allowing the use of standard statistical methods based on multivariate normality (Aitchison 1986). To facilitate calculation of log-ratios, zeros were replaced with a small value (for our data 0.01 % was appropriate, being two orders of magnitude below the smallest measured percentage; Aebischer et al. 1993). Then, across all individuals in each amphipod group, MANOVA compared food consumption to availability, testing the null hypothesis of random food consumption using Wilks’ lambda (Λ). Significance was determined by randomisation (*n* = 1999). Following a significant MANOVA, an electivity ranking was generated based on differences between consumption and availability (as log-ratios) for each pair of food types. Mean differences across individuals were used to rank food types in order of importance to amphipod diet, with significant rankings identified by randomisation (*n* = 1999, which generated stable ranking matrices).

## Results

### Functional response (FR) experiments

#### Predation of carp eggs and larvae

In experimental arenas, mortality of carp eggs (21.3 %) and carp larvae (50.4 %) was significantly greater than mortality in controls (0.0 and 3.2 % respectively; Fisher’s exact tests *p* < 0.001 for both), implying that amphipods were acting as predators rather than scavengers. Amphipods were also directly observed to prey upon live eggs and larvae. However, there was variation in predation rate between individuals, including some intermediate *D. villosus* and large *G. pulex* that consumed nothing even when presented with prey at the highest densities (Fig. S2).

**Fig. 1:**
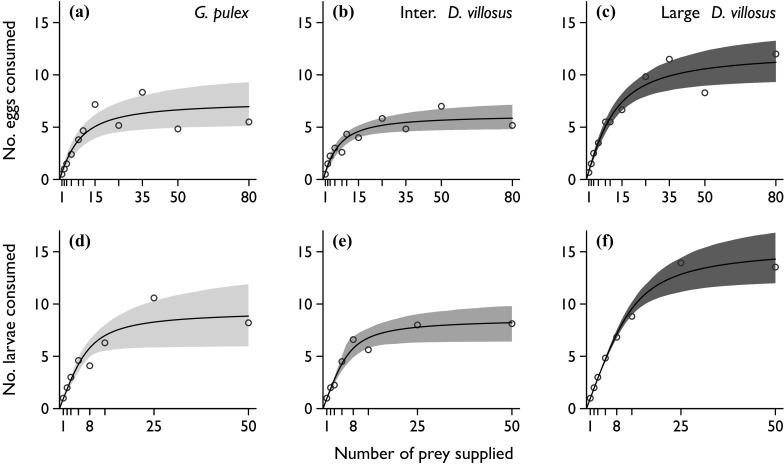
Rogers type II functional responses of amphipods on carp eggs (*upper three panels*) and carp larvae (*lower three panels*). Predators are *Gammarus pulex* (**a**, **d**), intermediate *Dikerogammarus villosus* (**b**, **e**) and large *D. villosus* (**c**, **f**). *Open circles* are means at each density supplied (*n* ≥ 4 for all prey densities and *n* ≥ 6 for prey densities of ten or above). *Shaded regions* are approximate 95 % confidence intervals for functional response curves based on 1999 bootstraps

FRs of all amphipod groups on both carp eggs and larvae were Type II (logistic regression first order coefficients significantly negative; Fig. 1, Table S2). Large *D. villosus* had a significantly shorter handling time on both eggs and larvae than the smaller amphipods, which did not differ in their handling time (Tables 2, 3). By inference, large *D. villosus* had a significantly higher maximum feeding rate (1/*h*T) on both carp eggs (12.3 day^−1^) and carp larvae (15.6 day^−1^) than the smaller amphipods (6.2 and 8.6 day^−1^ respectively for intermediate *D. villosus*, and 7.5 and 9.4 day^−1^ for *G. pulex*). The attack coefficient on eggs or larvae did not differ between the three amphipod groups (Tables 2, 3).

**Table 2:** Functional response parameter estimates for three amphipod groups on carp eggs and carp larvae as prey, extracted from Rogers’ random predator equation fitted to data in the *frair* package (Pritchard 2014)

Prey	Amphipod group	*a*	SE	*h*	SE	1/*h*T
Carp eggs	*G. pulex*	1.269	0.232	0.133	0.012	7.5
	Inter. *D. villosus*	1.419	0.343	0.162	0.016	6.2
	Large *D. villosus*	1.710	0.239	0.081	0.006	12.3
Carp larvae	*G. pulex*	3.410	0.910	0.107	0.012	9.4
	Inter. *D. villosus*	3.861	0.869	0.116	0.010	8.6
	Large *D. villosus*	4.115	0.638	0.064	0.004	15.6

*a* attack coefficient, *h* handling time (days.prey item^−1^), 1/*h*T maximum feeding rate (prey.day^−1^), where *T* time in days, *SE* standard error

**Table 3:** Comparison between functional response parameter estimates for three amphipod groups on carp eggs and carp larvae as prey, based on analysis using indicator variables in the *frair* package (Pritchard 2014)

Prey	Base group	Comparison		Estimate (*Da* or *Dh*)	SE	*z*	*p*
Carp eggs	Inter. *D. villosus*	*G. pulex*	*a*	−0.151	0.414	−0.365	0.715
			*h*	−0.028	0.020	−1.408	0.159
	Inter. *D. villosus*	Large *D. villosus*	*a*	0.290	0.418	0.694	0.488
			***h***	−0.080	0.171	−4.689	**<0.001**
	Large *D. villosus*	*G. pulex*	*a*	−0.441	0.333	−1.324	0.186
			***h***	0.052	0.014	3.839	**<0.001**
Carp larvae	Inter. *D. villosus*	*G. pulex*	*a*	−0.451	1.258	−0.358	0.720
			*h*	−0.009	0.016	−0.598	0.550
	Inter. *D. villosus*	Large *D. villosus*	*a*	0.251	1.079	0.233	0.816
			***h***	−0.052	0.011	−4.532	**<0.001**
	Large *D. villosus*	*G. pulex*	*a*	−0.709	1.110	−0.639	0.523
			***h***	0.042	0.013	3.321	**<0.001**

Significant differences (α = 0.05) are indicated in bold

*a* attack coefficient, *h* handling time (days.prey item^−1^), *D* difference, *SE* standard error

Every amphipod group had a significantly higher attack coefficient on carp larvae than on eggs. Handling times were also shorter on larvae than on eggs, but only significantly so for *D. villosus* (indicator variable comparisons on eggs as base and larvae as comparator: *G. pulex* difference in attack coefficient (*Da*) = 2.14, *p* = 0.023, difference in handling time (*Dh*) = −0.03, *p* = 0.114; intermediate *D. villosus Da* = 2.44, *p* = 0.009, *Dh* = −0.05, *p* = 0.017; large *D. villosus Da* = 2.41, *p* < 0.001, *Dh* = −0.02, *p* = 0.027).

Carp eggs were always completely consumed. Partial consumption of carp larvae was exhibited by individuals within all amphipod groups, but was rare and low in magnitude: only 34 % of amphipods partially consumed larvae, and amongst these the number of partially consumed larvae was low (mode = 1, median = 2, range 1–6). The incidence of partial consumption did not differ between amphipod groups (not retained in MAM) but was positively associated with prey density (binomial GLM *n* = 133, ф = 1.134, Deviance_1,131_ = 58.33, *p* < 0.001). Amongst amphipods that partially consumed larvae, number of partially consumed larvae increased with prey density with marginal significance (quasipoisson GLM *n* = 45, ф = 0.69, Deviance_1,43_ = 2.55, *p* = 0.061) whilst proportional partial consumption significantly decreased with increasing prey density (quasibinomial GLM *n* = 45, ф = 0.59, Deviance_1,43_ = 21.62, *p* < 0.001). Neither the number nor proportion of available larvae that were partially consumed differed between amphipod groups (not retained in MAMs). The similarity in partial consumption between amphipod groups, in addition to its rarity and low magnitude, means it did not decouple predatory consumption from killing and likely population impact: separate analyses of prey killed reveal identical patterns to analyses of prey consumed (Section S3, Supplementary Information).

#### Predation of trout eggs and larvae

In experimental arenas, mortality of trout larvae was low (4.5 %), but exceeded mortality in controls (2.2 %; Fisher’s exact test *p* = 0.022) implying that amphipods were preying upon trout larvae. As further evidence of predation, live but damaged larvae were observed in some arenas at the end of experiments, and in separate arenas amphipods were directly observed to prey upon live trout larvae.

**Fig. 2:**
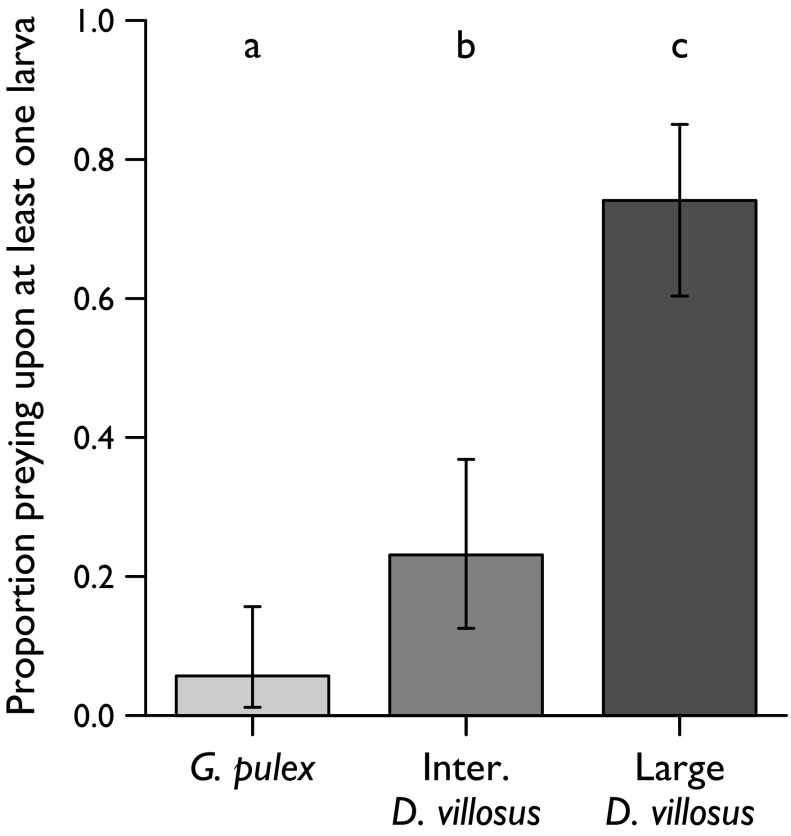
Proportion of each amphipod group that preyed upon (killed) trout larvae in functional response experiments (*n G. pulex* = 53, *n* intermediate *D. villosus* = 52, *n* large *D. villosus* = 54). *Error bars* are 95 % Clopper–Pearson confidence intervals. *Letters* indicate significant differences based on a binomial GLM

Only 3 of 53 *G. pulex*, 12 of 52 intermediate *D. villosus* and 40 of 54 large *D. villosus* preyed upon trout larvae. This incidence of predation did not depend on prey density (not retained in MAM) but significantly differed between amphipod groups (Fig. 2; binomial GLM *n* = 159, ф = 1.02, Deviance_2,156_ = 64.03, *p* < 0.001). Large *D. villosus* were more likely to kill trout larvae than intermediate *D. villosus* (*z* = 4.98, *p* < 0.001), which in turn were more likely do so than *G. pulex* (*z* = 2.37, *p* = 0.018). Amongst the amphipods that preyed upon trout larvae, the magnitude of predation was low (mode and median number of larvae killed = 1, maximum = 2), although this did not differ between amphipod groups or depend on prey density (neither explanatory variable retained in MAM).

Partial consumption of killed larvae was frequent, but with no evidence of differing incidence across amphipod groups or prey densities (neither explanatory variable retained in MAM). Of the larvae attacked by intermediate *D. villosus*, 86 % were partially consumed, compared to 70 % of larvae attacked by large *D. villosus* and 67 % of larvae attacked by *G. pulex*. The high incidence of partial consumption decoupled killing from feeding. Thus, despite no difference between amphipod groups in number of prey *killed* by predators, amphipod groups differed in the amount of larval flesh *consumed* by predators (Kruskal–Wallis χ^2^ = 7.25, *df* = 2, *p* = 0.027). Large *D. villosus* consumed a greater amount of the larvae they killed (median 0.80 larvae, interquartile range 0.50) than intermediate *D. villosus* (median 0.25, interquartile range 0.33; Dunn test adjusted *p* = 0.015). Consumption by *G. pulex* was not significantly different to consumption by either size class of *D. villosus*, but this is influenced by the small sample size for *G. pulex* (three individuals consumed 0.2, 0.2 and 1.0 larvae respectively).

Incidence of predation on trout eggs was even lower than on trout larvae. Trout eggs were completely consumed by only 3 of 152 amphipods: two large *D. villosus* and one *G. pulex*. Burst eggs were occasionally observed in tanks at the end of experiments and some of the openings appeared to have been nibbled. However, we make no further analysis of this damage (a) because it occurred rarely, (b) a very small proportion (c. 5 %) of each damaged egg was apparently consumed and (c) because bursting did not occur any more frequently in tanks with amphipods (0.6 % of eggs burst) compared to control tanks (0.9 %; Fisher’s exact test *p* = 0.529), so initial bursting (and death) of the egg is unlikely to have been caused by the amphipods.

### Electivity experiments

In electivity experiments, consumption of eggs and larvae was assumed to reflect amphipod predation because mortality in control arenas was very low (eggs 0.8 %, larvae 0.0 %) and no partial consumption of eggs or larvae was observed in experimental arenas. Mortality of *A. aquaticus* in control arenas was also low (3.4 %).

**Fig. 3:**
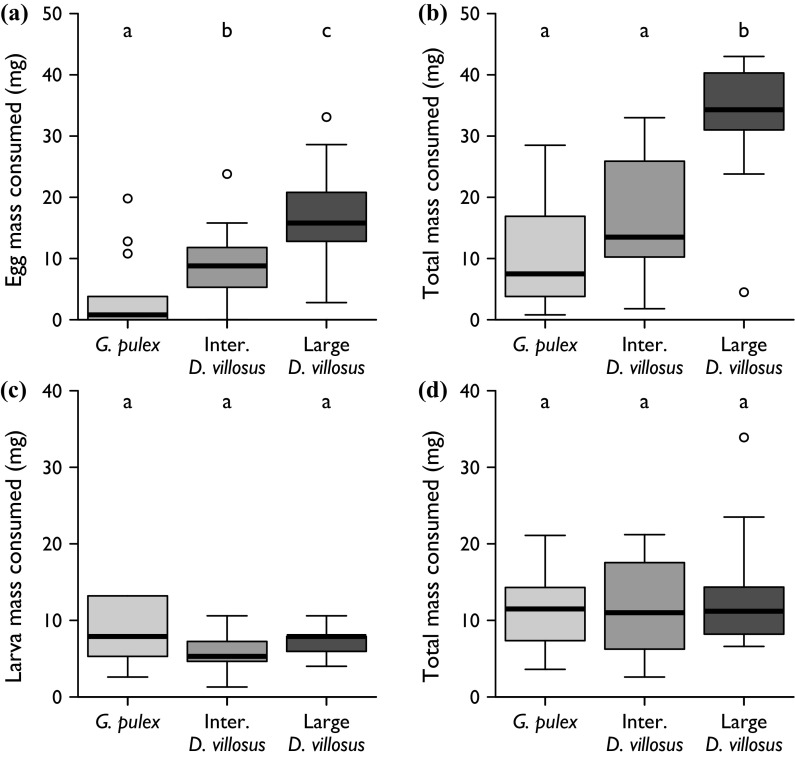
Consumption of food by each amphipod group used in electivity experiments involving carp eggs (**a**, **b**) or carp larvae (**c**, **d**). *Panels* on *left* (**a**, **c**) show consumption of the focal fish prey, whilst *panels* on *the right* (**b**, **d**) show total consumption of all food types combined. Masses are adjusted for autogenic change. *Boxes* show medians and interquartile ranges; *whiskers* indicate data range; *circles* are outliers. *Letters above boxes* indicate significant differences based on Tukey HSD or Dunn post hoc tests, as appropriate to each data set. *n* ≥ 9 for all boxes: precise samples sizes are given in Fig. 4

In electivity experiments involving carp eggs, the amphipod groups consumed different masses of eggs (Fig. 3a; Kruskal–Wallis χ^2^ = 15.20, *df* = 2, *p* < 0.001). *D. villosus* consumed a greater mass of eggs than size-matched *G. pulex* (Dunn test adjusted *p* = 0.020) and large *D. villosus* consumed a greater mass of eggs than intermediate *D. villosus* (Dunn test adjusted *p* = 0.035). This is partially explained by differences in overall consumption (Fig. 3b; ANOVA *F*_2,36_ = 13.05, *p* < 0.001). Large *D. villosus* ate more food in total than intermediate *D. villosus* (Tukey HSD *p* = 0.004) and *G. pulex* (Tukey HSD *p* < 0.001). The size-matched amphipods did not differ in the amount of food consumed (Tukey HSD *p* = 0.157) although there was a tendency for *D. villosus* to consume more (Fig. 3b).

**Fig. 4:**
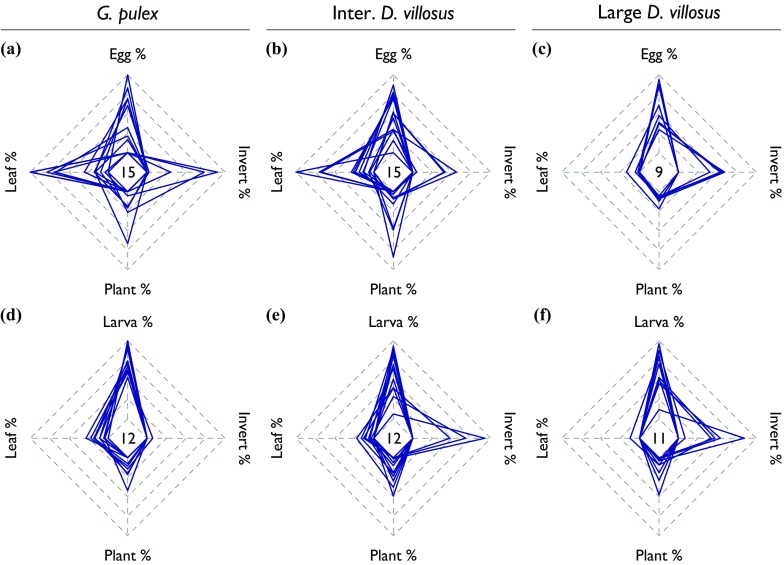
*Radar plots* representing the diet compositions of amphipods in experiments involving carp eggs (*upper three panels*) and carp larvae (*lower three panels*). Amphipods are *Gammarus pulex* (**a**, **d**), intermediate *Dikerogammarus villosus* (**b**, **e**) and large *D. villosus* (**c**, **f**). For each experiment-amphipod combination, *n* is given in the centre of the respective plot. The diet of each individual amphipod is represented by a *dark blue polygon*, with each vertex representing the percentage of each of the four food types in the diet of that amphipod; note that some polygons overlap. Plots constructed in package *fmsb* (Nakazawa 2015)

Amongst considerable inter-individual variation in diet composition, each amphipod group overall fed non-randomly in electivity experiments involving eggs (Fig. 4a–c; *G. pulex* Wilks’ Λ = 0.52, *p* = 0.046; intermediate *D. villosus* Λ = 0.26, *p* = 0.002; large *D. villosus* Λ = 0.06, *p* = 0.007). Eggs made the greatest contribution to *D. villosus* diet (Table 4), reflecting the fact that most individuals consumed eggs (100 % of large *D. villosus* and 93 % of intermediate *D. villosus*) and eggs made up the majority of *D. villosus* diet, on average (58 % of large and 50 % of intermediate). *Large D. villosus* supplemented egg predation with herbivory (plant material was consumed by all individuals but in small amounts) or predation on *A. aquaticus* (making a large contribution to individual diet but for only 56 % of individuals). Intermediate *D. villosus* supplemented egg predation with detritivory: leaf material was consumed by 73 % of individuals and made up 25 % of the diet on average. In contrast, leaf material was at the top of the electivity ranking for *G. pulex*, being consumed by 87 % of individuals and constituting 47 % of the diet on average. Unlike *D. villosus*, the native amphipods did not consume eggs significantly more or less than any other food item (Table 4). Only 54 % of *G. pulex* individuals consumed eggs, and eggs constituted on average 30 % of *G. pulex* diet.

**Table 4:** Ranking of food types by contribution to amphipod diet, based on a comparison of percentage consumption to percentage availability (Aebischer et al. 1993; Calenge 2015)

Expt	Contribution rankings					
	*G. pulex*		Inter. *D. villosus*		Large *D. villosus*	
Eggs	Leaf	a	Egg	a	Egg	a
	Egg	ab	Leaf	ab	Plant	b
	Plant	b	Plant	bc	Invert	abc
	Invert	b	Invert	c	Leaf	c
Larvae	Larva	a	Larva	a	Larva	a
	Leaf	b	Plant	b	Plant	b
	Plant	b	Leaf	b	Invert	bc
	Invert	c	Invert	b	Leaf	c

Full ranking matrices are given in Table S4. Eggs or larvae were presented alongside the other food items in separate experiments (Expts). Invert–invertebrate (*Asellus aquaticus*)

When carp larvae were presented as one of the food options, feeding by the three amphipod groups was remarkably similar. There was no difference in the mean mass of larvae consumed by predators in each group (Fig. 3c; ANOVA *F*_2,32_ = 2.32, *p* = 0.115) or in the log-transformed mean mass of all food consumed (Fig. 3d; ANOVA *F*_2,32_ = 0.45, *p* = 0.639).

Again, each amphipod group fed non-randomly in electivity experiments with larvae as prey (Fig. 4d–f; *G. pulex* Λ = 0.04, *p* = 0.001; intermediate *D. villosus* Λ = 0.07, *p* = 0.001; large *D. villosus* Λ = 0.03, *p* = 0.001). Larvae made the greatest contribution to the diet of all amphipod groups (Table 4): all amphipods consumed larvae and larvae formed the greatest proportion of diets, especially for *G. pulex* (on average 78 % *G. pulex* diet was carp larvae, compared to 60 % for intermediate *D. villosus* and 66 % for large *D. villosus*). The amphipod groups differed in the food they consumed to supplement larval predation. For example, large *D. villosus* tended to consume plant and invertebrate material as above, whilst *G. pulex* consumed leaf and plant material and avoided *A. aquaticus* (Fig. 4d, f).

## Discussion

The ‘killer shrimp’ *D. villosus* is spreading across Europe with significant ecological impacts, including declines in resident macroinvertebrate populations attributed to predation by the invader (Dick and Platvoet 2000; Josens et al. 2005; van Riel et al. 2006; MacNeil et al. 2013). Since *D. villosus* has been observed to feed upon fish eggs and larvae, there is concern over its potential impact on biologically and commercially important fish populations. One major contributor to impact is *per capita* effect (Parker et al. 1999) and our data suggest invasive *D. villosus* will have a greater *per capita* effect than native *G. pulex* on fish populations as a predator of eggs and larvae. However, this is more a reflection of the larger size of the invader (pers. obs.; Pinkster 1970; Nesemann et al. 1995) than any intrinsic interspecific difference in predation. Relative to the smaller amphipods, large *D. villosus* showed (a) a greater consumption of food per se (b) a greater tendency to consume animal prey, including fish eggs and larvae, and (c) greater ability to prey upon larger fish eggs and larvae.

Large amphipods consume food (of a given size) at a greater rate than small amphipods. In FR experiments, maximum feeding rates of large *D. villosus* were 1.6 and 1.7 times greater than *G. pulex* on carp eggs and larvae respectively, and 2.0 and 1.8 times greater than intermediate *D. villosus*. These differences reflect the shorter handling times of large *D. villosus* on both prey types. In experiments with trout larvae, large *D. villosus* also consumed a greater mass of the trout larvae they killed than did intermediate *D. villosus*. In electivity experiments with carp eggs, large *D. villosus* consumed the most eggs and the most food in total: median 4.6 times more food than *G. pulex* and 2.5 times more food than intermediate *D. villosus*.

Anomalously, in electivity experiments with carp larvae, large *D. villosus* consumed a similar mass of food and larvae as the smaller amphipods. The low consumption of larvae probably reflects an interaction between predator size, prey type and substrate. The largest amphipods are less able to manoeuvre through interstitial spaces, but motile prey can make best use of these spaces to evade predation (Barrios-O’Neill et al. 2015). However, it is not clear why low consumption of larvae should be associated with low overall consumption i.e. why large *D. villosus* did not consume other food items in larger quantities to compensate.

The generally positive association between size and resource consumption is in accord with previous empirical work with amphipods (Maier et al. 2011; Dodd et al. 2014) and, given the predator–prey body size ratios in the present experiment, more general theoretical work (Brose 2010; Rall et al. 2012). Metabolic rate scales positively with size (Kleiber 1932). This fundamental physiological difference must be balanced by higher consumption rates in larger amphipods, facilitated by morphological differences such as larger mouthparts and a larger gut volume which decrease the time needed to subdue, ingest and digest prey of a given size (Brose 2010; Vucic-Pestic et al. 2010). The similarity of attack coefficients across all three amphipod groups suggests that such physiological and morphological factors, rather than behavioural ones, determine the higher feeding rate of large *D. villosus*. However, we acknowledge that the lack of differentiation in attack coefficients could be an artefact of the non-replacement design of our FR experiments (Dick et al. 2014).

As well as consuming more *per se*, large amphipods are more predatory than smaller amphipods. Whilst all amphipod groups were omnivorous in electivity experiments, in accord with MacNeil et al. (1997) and with potential fitness benefits (Cruz-Rivera and Hay 2000), animal prey tended to make a greater contribution to the diet of large *D. villosus*. It was the only amphipod group for which eggs and larvae were consumed significantly more than all other food types, and for which invertebrates (*A. aquaticus*) were not rooted at the bottom of the diet-contribution rankings. Size-based dietary shifts in *D. villosus* are also apparent in the field, with stable isotope analyses indicating a tendency for large individuals to be more predatory (van Riel et al. 2006; Koester et al. 2016). It is likely that this predatory tendency will be directed towards fish eggs and larvae in the field, given the tendency of *D. villosus* to consume eggs over alternative prey (this paper; Casellato et al. 2007) and general electivity towards benthic prey (Dodd et al. 2014).

Larger predators are also able to capture and kill larger prey than small predators (Elton 1927; Woodward et al. 2005; Brose 2010). By virtue of their size and associated massive mouthparts, large *D. villosus* are better equipped to kill large prey. *D. villosus* can therefore have a greater impact on fish species with large eggs and larvae, such as salmonids—which were almost invulnerable to *G. pulex* predation in our experiments. Further, the ability to feed on larger prey could intensify the impact of *D. villosus* on any given fish species in the field, given that it will be able to prey upon fish larvae for a longer period: it will take larvae longer to grow to a size that is invulnerable to *D. villosus* predation.

Meanwhile, size-matched *D. villosus* and *G. pulex* had similar predatory impacts. Neither could prey upon trout eggs, they consumed similar a similar mass of carp larvae in electivity experiments, and incidence and magnitude of partial consumption were comparable between the species. Most strikingly, FRs on both carp eggs and larvae did not differ between the size-matched amphipods—in terms of shape, attack coefficients, handling times or maximum feeding rates. Type II FRs are consistent with published amphipod FRs on invertebrate prey (Bollache et al. 2008; Alexander et al. 2012; Dodd et al. 2014; Médoc et al. 2015). The similarity of FR parameters probably reflects the nature of the prey (Moustahfid et al. 2010). Carp eggs and larvae are relatively soft, and predation rates of size-matched *D. villosus* and *G. pulex* tend to be similar on soft-bodied prey e.g. chironomid larvae (Krisp and Maier 2005; Dodd et al. 2014). Pronounced differences between feeding rates occur when the prey is relatively tough e.g. *A. aquaticus* (Bollache et al. 2008; Dodd et al. 2014).

There were, however, two subtle differences between the size-matched amphipod species. Both are associated with a higher predatory impact of *D. villosus*, complementing its size-based impact, but are smaller in magnitude than differences related to size, so are likely to play a much smaller role in dictating impacts in the field. First, *D. villosus* was more likely than *G. pulex* to prey upon trout larvae, perhaps because its long gnathopods aid handling of large prey (Mayer et al. 2009) or its higher glycogen reserves facilitate high-speed attacks to counter defensive burst swimming (Maazouzi et al. 2011). Secondly, *G. pulex* consumed fewer carp eggs than *D. villosus* in electivity experiments. *G. pulex* may be less able to crush or puncture egg capsules than *D. villosus*, and thus rejects eggs in favour of soft decaying leaves—but does not face this issue with softer carp larvae. Alternatively, the presence of habitat structure could have interfered with the detection of static carp eggs, but not motile larvae, by *G. pulex*.

In our experiments, coarse fish eggs and larvae were much more vulnerable to predation by amphipods than salmonid eggs and larvae. Whilst carp eggs were readily consumed, trout eggs were almost completely invulnerable to amphipod predation and few amphipods, of any size, killed more than one trout larva over 48 h. These differences in predation could reflect differences in prey size, defensive mechanisms, and/or temperature. Trout eggs and larvae are larger than those of carp. Consequently, predator–prey body size ratios of amphipods to salmonid larvae are very low (e.g. 0.45 for large *D. villosus* and trout larvae) and at these ratios attack rates are low and handling times long (Luecke et al. 1990; Brose 2010; Rall et al. 2012). Each individual salmonid larva also presents a large mass of food to be processed, meaning they will take a long time to consume and fewer individual larvae will be needed to induce predator satiation. In addition, trout eggs and larvae are both more physically defended than their coarse counterparts. Trout larvae are strong burst swimmers, assisting them to evade capture (Fuiman 2002). Trout eggs possess a thick, tough outer casing (chorion) to protect them from mechanical damage when buried in redds (Zotin 1958), but the chorion could also provide an important defensive mechanism against biological enemies such as fungal diseases (Songe et al. 2016) and invertebrate egg predators (this paper). Finally, the difference in predatory impact may also reflect differences in temperature. We conducted our experiments in temperatures around which trout (7 °C) and carp (14 °C) eggs develop in the field (Alabaster and Lloyd 1982). As ectotherms, amphipod metabolism and activity—including predation—will likely be reduced at lower temperatures (Sutcliffe et al. 1981; van der Velde et al. 2009; Maier et al. 2011). Low *per capita* predation rates on trout larvae do not negate the potential for substantial mortality in the field, however. Daily predation will accumulate over the long development period of salmonid eggs and larvae (Teletchea and Fontaine 2010), and salmonids have a relatively small reproductive output (Winemiller and Rose 1992), which increases the importance of each individual larva to the population.

In addition to its higher *per capita* effect by virtue of its large size, the impact of *D. villosus* in the field may be further magnified by its abundance (Parker et al. 1999; Ricciardi 2003). *D. villosus* reaches locally high densities (up to 10,000 m^−2^; van Riel et al. 2006) which may exceed those of other amphipods in comparable systems. In the River Meuse, for example, invading *D. villosus* accumulates to higher densities (200–500 individuals per artificial substrate) than the previous native-naturalised community (50–120 individuals per substrate), of which *G. pulex* was part (Josens et al. 2005). This conforms to the general pattern of aquatic invasive species reaching higher densities, on average, than native analogues (Hansen et al. 2013). Although *per capita* effects may increase nonadditively with density as a result of interference between conspecifics (Hassell 1978; Médoc et al. 2015), increased densities will be associated with increased impact provided this multiple predator effect is not antagonistic. Moreover, the larger size of *D. villosus* means more individuals within the population will exceed the (unquantified) size threshold at which amphipods can feed on fish eggs and larvae (cf. Mills 1981). Consequently, a greater proportion of individuals within *D. villosus* populations will be acting as predators—so differential abundance of *predators* will be even greater than apparent from a comparison of total abundance.

It is possible that the high density and biomass of *D. villosus* could somewhat offset its negative effects as a predator. It has been suggested that this invasive amphipod will provide a plentiful food resource for fish that traverse the predatory gauntlet (Luecke et al. 1990) to reach adulthood, perhaps boosting survival and fecundity (Kelleher et al. 1998; Madgwick and Aldridge 2011; Brandner et al. 2013; Czarnecka et al. 2014). However, the higher density of *D. villosus* could just compensate for its lower quality and profitability as prey (Arbaciauskas et al. 2010; Błońska et al. 2015) and so provide little additional benefit to fish populations.

On balance, the high *per capita* effect and high density of *D. villosus* indicate it may have a stronger negative impact on fish populations, through predation of eggs and larvae, than the native *G. pulex* it is likely to replace (Dick and Platvoet 2000)—although this impact is context-dependent and could vary in space and time (Ricciardi 2003). Where *D. villosus* imposes even a small additive increase in mortality, recruitment into fish populations could be significantly reduced. In fish, small changes in the slope of the survivorship curve in the early life stages can coarsely control a cohort’s abundance later in life (Bagenal and Braum 1968; Houde 2002). In this context, both coarse fish and salmonid populations could be negatively affected by *D. villosus* invasion: in both cases, the predatory impact of *D. villosus* is *greater* than that of native *G. pulex*. Reduced recruitment could be particularly detrimental to populations of the 37 % of European freshwater fish species that are already threatened (Freyhof and Brooks 2011). Furthermore, reduced recruitment to populations exploited by anglers could negatively impact this economically and socially valuable activity (Mawle and Peirson 2009; Brown et al. 2012). Although some commercial fish populations are maintained entirely by stocking of post-larval fish and will be unaffected by amphipod predation, populations that depend at least partly on natural recruitment could be suffer under the additional mortality imposed by *D. villosus*. Fish densities will be reduced or supplementary stocking, and its associated expenditure, must be increased to compensate.

Understanding and management of invasive species will be improved by the availability of quantitative evidence of their impacts (NRC 2002; Sutherland et al. 2004; Kumschick et al. 2012). Our laboratory experiments contribute to this evidence for *D. villosus*, suggesting this invader will have a greater negative impact on fish populations than native *G. pulex* through predation on eggs and larvae. The higher *per capita* impact of *D. villosus* on fish is primarily due to its larger body size. Thus, in this system—and for predicting invasive species’ impacts in general—size matters.

## Electronic supplementary material

Below is the link to the electronic supplementary material.

Supplementary material 1 (DOCX 127 kb)
