# Supplementary material for: Size matters: predation of fish eggs and larvae by native and invasive amphipods
Source: Biol Invasions. 2016 Sep 8;19(1):89–107. doi: 10.1007/s10530-016-1265-4 (PMC7175601; doi:10.1007/s10530-016-1265-4)
Supplement: Supplementary file 1 — Supplementary material 1 (DOCX 127 kb) [file 10530_2016_1265_MOESM1_ESM.docx]

**Supplementary Information**

for Taylor, N.G. & Dunn, A.M.

*Size matters: predation of fish eggs and larvae by native and invasive amphipods*

Correspondence: a.dunn@leeds.ac.uk

**1. Amphipod sizes**

Masses and lengths of amphipods used in each experiment (combination of experimental design, fish species and developmental stage in Table S1) were compared using ANOVAs. Length and mass were log-transformed where necessary to conform to model assumptions. Pairwise post-hoc comparisons were made using Tukey HSD tests.

ANOVAs for both length and mass were significant (ANOVA *p* < 0.001) for all experiments. Post-hoc tests confirmed that across all experiments, large *D. villosus* were significantly heavier and longer than both *G. pulex* and intermediate *D. villosus* (Tukey HSDs *p* < 0.001 for all tests). *G. pulex* and intermediate *D. villosus* did not differ in mass or length in any experiment (Tukey HSDs *p* > 0.43 in all tests except for comparison of length in carp larvae functional response experiment *p* = 0.081).

| **Table S1** Size (mass and length) of amphipods used in each experiment. Amphipods were blotted dry before measurement of mass; lengths are from rostrum tip to telson tip for amphipods in natural, curved resting state. In all experiments, large *D. villosus* is significantly larger than both large *G. pulex* and intermediate *D. villosus,* which in turn do not differ significantly in size. | | | | | | | | | |
| --- | --- | --- | --- | --- | --- | --- | --- | --- | --- |
| **Experimental Design** | **Prey sp.** | **Stage** | **Amphipod Mass (mg) or Length (mm)** | **Large**  ***G. pulex*** | | **Intermediate**  ***D. villosus*** | | **Large**  ***D. villosus*** | |
|  |  |  |  | Mean | SE | Mean | SE | Mean | SE |
| Functional Response | Carp | Eggs | Mass | 52.3 | 1.0 | 54.0 | 1.2 | 105.0 | 1.7 |
|  |  |  | Length | 17.2 | 0.1 | 17.5 | 0.2 | 22.1 | 0.1 |
|  |  | Larvae | Mass | 51.7 | 1.0 | 53.9 | 1.3 | 106.7 | 2.0 |
|  |  |  | Length | 17.1 | 0.1 | 17.6 | 0.2 | 22.3 | 0.2 |
|  | Trout | Eggs | Mass | 41.4 | 1.0 | 42.7 | 1.2 | 109.5 | 2.8 |
|  |  |  | Length | 17.2 | 0.1 | 17.5 | 0.2 | 22.1 | 0.1 |
|  |  | Larvae | Mass | 41.4 | 0.8 | 43.2 | 1.2 | 106.1 | 3.0 |
|  |  |  | Length | 15.9 | 0.1 | 15.9 | 0.2 | 21.6 | 0.3 |
| Electivity | Carp | Eggs | Mass | 49.0 | 2.3 | 53.3 | 3.4 | 102.1 | 4.1 |
|  |  |  | Length | 16.9 | 0.3 | 17.3 | 0.4 | 21.9 | 0.3 |
|  |  | Larvae | Mass | 49.4 | 1.9 | 50.9 | 3.5 | 110.8 | 4.3 |
|  |  |  | Length | 16.7 | 0.3 | 16.2 | 0.6 | 23.1 | 0.4 |

We also compared a combined index of body size, derived from principal components analysis on log length and log mass, amongst amphipod groups in each experiment. The first principal component described between 96.7 and 98.6% of the variance in body size. Results based on this first principal component confirmed the previous analysis on length and mass separately: large *D. villosus* were bigger than the other amphipod groups in all experiments (Tukey HSDs *p* < 0.001), whilst *G. pulex* and intermediate *D. villosus* did not differ in body size (Tukey HSDs *p* > 0.52 in all tests except for comparison of body size in carp egg experiment *p* = 0.152).

*G. pulex* and intermediate *D. villosus* were slightly larger in the carp experiments than the trout experiments, presumably due to seasonal differences in size structure of source amphipod populations (ANOVAs for amphipods used in egg experiments: *G. pulex* mass *F*_1,104_ = 59.1, *p* < 0.001 and length *F*_1,104_ = 30.1, *p* < 0.001; intermediate *D. villosus* mass *F*_1,106_ = 42.2, *p* < 0.001 and length *F*_1,106_ = 19.2, *p* < 0.001). Large *D. villosus* did not differ in size between seasons (ANOVAs for amphipods used in egg experiments: mass *F*_1,120_ = 2.05, *p* = 0.155 and length *F*_1,120_ = 1.55, *p* = 0.216) but this could reflect deliberate selection of similar-sized individuals rather than the actual maximum sizes within the population.

**2. Functional response analyses on prey consumed** (additional information)

| **Table S2** Parameter estimates and significance levels from second order logistic regression of the proportion of prey eaten against initial prey density, for three amphipod groups (AGs). Quasibinomial errors were used due to overdispersion. ф – dispersion parameter for GLM; N_0_ – first order term; N^2^_0_ – second order term; *Gp* – *G. pulex*; *Dv* In – intermediate *D. villosus*; *Dv* Lg – large *D. villosus*. | | | | | | | | | |
| --- | --- | --- | --- | --- | --- | --- | --- | --- | --- |
| **Prey** | **AG** | **ф** | **Intercept** | ***p*** | **N_0_** | ***p*** | **N^2^_0_** | ***p*** | **Type** |
| Carp eggs | *Gp* | 3.975 | 0.471 | 0.260 | -0.067 | 0.006 | 3.536 x 10^-4^ | 0.185 | II |
|  | *Dv* In | 1.371 | 0.238 | 0.337 | -0.067 | < 0.001 | 3.921 x 10^-4^ | 0.016 | II |
|  | *Dv* Lg | 1.923 | 1.157 | < 0.001 | -0.079 | < 0.001 | 5.323 x 10^-4^ | <0.001 | II |
| Carp larvae | *Gp* | 2.318 | 1.675 | 0.003 | -0.107 | 0.018 | 8.428 x 10^-4^ | 0.239 | II |
|  | *Dv* In | 1.929 | 2.312 | < 0.001 | -0.176 | < 0.001 | 1.956 x 10^-3^ | 0.008 | II |
|  | *Dv* Lg | 1.185 | 3.147 | < 0.001 | -0.156 | < 0.001 | 1.484 x 10^-3^ | 0.006 | II |


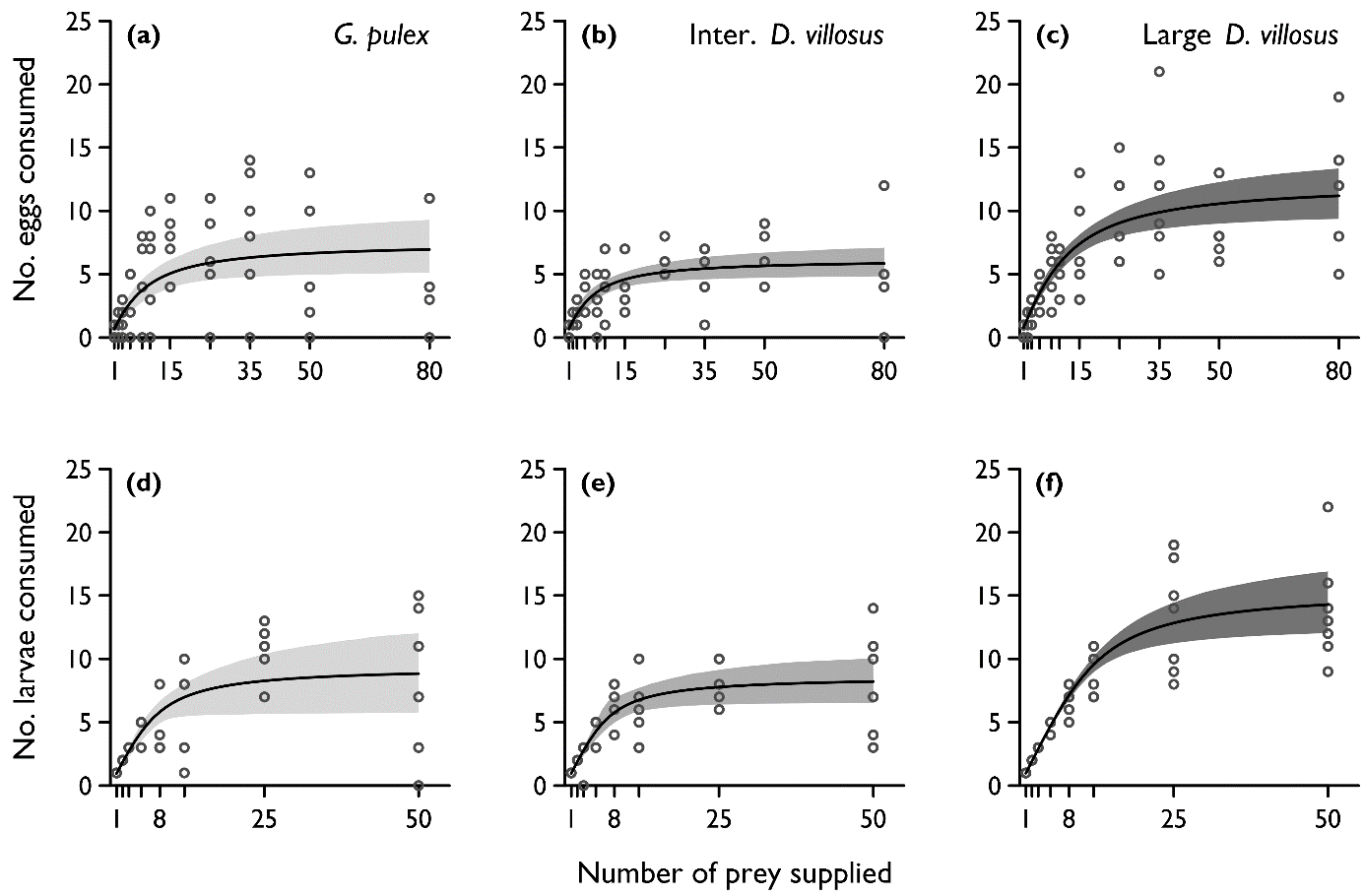


**Fig. S2** Functional response fits in the context of individual data points, shown as open circles (note some points represent more than one individual; *n* ≥ 4 for all prey densities and *n* ≥ 5 for prey densities of five or above). Fitted curves are Rogers Type II functional responses with bootstrapped 95% confidence intervals (*n* = 1999). Upper three panels refer to consumption of carp eggs, lower three panels consumption of carp larvae. Predators are *Gammarus pulex* (a,d), intermediate *Dikerogammarus villosus* (b,e) and large *D. villosus* (c,f).

**3. Functional response analyses on larvae killed** (rather than consumed)

In functional response experiments, some partial consumption of carp larvae was observed. When considering impacts of predators on prey populations, it is the number of prey killed (rather than consumed) which is important. If partial consumption is common relative to complete consumption, killing is less strongly related to satiation and the link between consumptive FRs and population impact is weakened (Dick et al. 2002). The carp larvae FR data were re-analysed using number of prey killed as response variables. The number of larvae killed was calculated as the number of larvae supplied minus the total number of live or dead but undamaged larvae remaining. These analyses yielded qualitatively identical and quantitatively similar results (Tables S3.1 to 3.3) to analyses based on prey consumption (see main paper). Thus, in this case consumptive FRs may provide a reasonable tool to infer impacts on prey populations.

| **Table S3.1** Parameter estimates and significance levels from second order logistic regression of the proportion of carp larvae killed against initial larval density, for three amphipod groups (AGs). Quasibinomial errors were used due to overdispersion. ф – dispersion parameter for GLM; N_0_ – first order term; N^2^_0_ – second order term; *Gp* – *G. pulex*; *Dv* In – intermediate *D. villosus*; *Dv* Lg – large *D. villosus*. | | | | | | | | | |
| --- | --- | --- | --- | --- | --- | --- | --- | --- | --- |
| **Prey** | **AG** | **ф** | **Intercept** | ***p*** | **N_0_** | ***p*** | **N^2^_0_** | ***p*** | **Type** |
| Carp larvae | *Gp* | 2.273 | 1.661 | 0.002 | -0.105 | 0.018 | 8.145 x 10^-4^ | 0.243 | II |
|  | *Dv* In | 2.001 | 2.265 | <0.001 | -0.169 | < 0.001 | 1.852 x 10^-3^ | 0.011 | II |
|  | *Dv* Lg | 1.192 | 3.069 | <0.001 | -0.150 | < 0.001 | 1.412 x 10^-3^ | 0.007 | II |

| **Table S3.2** Functional response parameter estimates for three amphipod groups on carp larvae as prey, extracted from Rogers’ random predator equation fitted to data in the *frair* package (Pritchard 2014).  *a* – attack coefficient; *h* – handling time (days.prey item^-1^); 1/*h*T – maximum feeding rate (prey.day-^1^), where T = time in days; SE – standard error. | | | | | | |
| --- | --- | --- | --- | --- | --- | --- |
| **Prey** | **Amphipod Group** | ***a*** | **SE** | ***h*** | **SE** | **1/*h*T** |
| Carp larvae | *G. pulex* | 3.424 | 0.854 | 0.100 | 0.011 | 10.0 |
|  | Inter. *D. villosus* | 3.643 | 0.796 | 0.107 | 0.010 | 9.3 |
|  | Large *D. villosus* | 3.757 | 0.539 | 0.058 | 0.004 | 17.4 |

| **Table S3.3** Comparison between functional response parameter estimates for three amphipod groups on carp larvae as prey, based on analysis using indicator variables in the *frair* package (Pritchard 2014). Significant differences (α = 0.05) are indicated in bold. *a* – attack coefficient; *h* – handling time (days.prey item^-1^); *D* – difference; SE – standard error. | | | | | | | |
| --- | --- | --- | --- | --- | --- | --- | --- |
| **Prey** | **Base** | **Comparison** |  | **Estimate**  **(*Da* or *Dh*)** | **SE** | ***z*** | ***p*** |
| Carp larvae | Inter. *D. villosus* | *G. pulex* | *a* | -0.222 | 1.167 | -0.190 | 0.850 |
|  |  |  | *h* | -0.007 | 0.014 | -0.489 | 0.625 |
|  | Inter. *D. villosus* | Large *D. villosus* | *a* | 0.114 | 0.962 | 0.118 | 0.906 |
|  |  |  | ***h*** | **-0.050** | **0.011** | **-4.628** | **<0.001** |
|  | Large *D. villosus* | *G. pulex* | *a* | -0.333 | 1.010 | -0.330 | 0.742 |
|  |  |  | ***h*** | **0.042** | **0.012** | **3.654** | **<0.001** |

**4. Compositional analysis: ranking matrices**

| **Table S4** Ranking matrices generated by compositional analysis (Aebischer et al. 1993; Calenge 2015), ranking food types on their proportional contribution to amphipod diets. Single symbol (+ or -) indicates a food type is ranked above or below another food type respectively. Triple symbols (+++ or ---) indicate this ranking is significant. Matrices were generated by randomisation (with *n* = 1999 generating the stable matrices presented). Analyses (i) assuming equal availability of food types and (ii) using actual proportional masses available yielded identical matrices. | | | | | | | | | | | | | | | | | | |
| --- | --- | --- | --- | --- | --- | --- | --- | --- | --- | --- | --- | --- | --- | --- | --- | --- | --- | --- |
|  |  | **CONSUMER: *G. pulex*** | | | | |  | **CONSUMER: Inter. *D. villosus*** | | | | |  | **CONSUMER: Large *D. villosus*** | | | | |
|  |  |  |  |  |  |  |  |  |  |  |  |  |  |  |  |  |  |  |
| **EGG experiments** |  |  | leaf | egg | plant | invert |  |  | egg | leaf | plant | invert |  |  | egg | plant | invert | leaf |
|  |  | leaf | 0 | + | +++ | +++ |  | egg | 0 | + | +++ | +++ |  | egg | 0 | +++ | + | +++ |
|  |  | egg | - | 0 | + | + |  | leaf | - | 0 | + | +++ |  | plant | --- | 0 | + | +++ |
|  |  | plant | --- | - | 0 | + |  | plant | --- | - | 0 | + |  | invert | - | - | 0 | + |
|  |  | invert | --- | - | - | 0 |  | invert | --- | --- | - | 0 |  | leaf | --- | --- | - | 0 |
|  |  |  |  |  |  |  |  |  |  |  |  |  |  |  |  |  |  |  |
| **LARVA experiments** |  |  | larva | leaf | plant | invert |  |  | larva | plant | leaf | invert |  |  | larva | leaf | plant | invert |
|  |  | larva | 0 | +++ | +++ | +++ |  | larva | 0 | +++ | +++ | +++ |  | larva | 0 | +++ | +++ | +++ |
|  |  | leaf | --- | 0 | + | +++ |  | plant | --- | 0 | + | + |  | leaf | --- | 0 | --- | - |
|  |  | plant | --- | - | 0 | +++ |  | leaf | --- | - | 0 | + |  | plant | --- | +++ | 0 | + |
|  |  | invert | --- | --- | --- | 0 |  | invert | --- | - | - | 0 |  | invert | --- | + | - | 0 |

**5. Compositional analysis: test statistics assuming equal availability of food types**

| **Table S5** MANOVA tests for non-random food consumption, assuming equal availability of food types. As for the analyses using actual availability of food types (presented in main paper and in Table S4), *p* values were generated by randomisation with *n* = 1999. | | | | | | |
| --- | --- | --- | --- | --- | --- | --- |
|  | ***G. pulex*** | | **Inter. *D. villosus*** | | **Large *D. villosus*** | |
|  | Wilks’ Λ | *p* | Wilks’ Λ | *p* | Wilks’ Λ | *p* |
| **Experiments with eggs** | 0.518 | 0.049 | 0.261 | 0.004 | 0.070 | 0.006 |
| **Experiments with larvae** | 0.048 | < 0.001 | 0.104 | 0.002 | 0.049 | 0.002 |

**6. References**

Aebischer NJ, Robertson PA, Kenward RE (1993) Compositional analysis of habitat use from animal radio-tracking. Ecology 74:1313–1325.

Calenge C (2015) adehabitatHS: analysis of habitat selection by animals. R Package version 0.3.12. http://cran.r-project.org/package=adehabitatHS

Dick JTA, Platvoet D, Kelly DW (2002) Predatory impact of the freshwater invader *Dikerogammarus villosus* (Crustacea: Amphipoda). Can J Fish Aquat Sci 1084:1078–1084. doi: 10.1139/F02-074

Pritchard DW (2014) frair: functional response analysis in R. R Package version 0.4. http://cran.r-project.org/package=frair
